# MaizeDB – A Functional Genomics Perspective

**DOI:** 10.1002/cfg.157

**Published:** 2002-04

**Authors:** Mary Polacco, Ed Coe, Zhiwei Fang, Denis Hancock, Hector Sanchez-Villeda, Steve Schroeder

**Affiliations:** 1 USDA-ARS USA; 2 Department of Agronomy University of Missouri Columbia MO 65211 USA; 3 203 Curtis Hall Dept Agronomy University of Missouri Columbia MO 65211 USA

## Abstract

MaizeDB (http://www.agron.missouri.edu/) has existed since the early 90’s as a genomespecific database that is grounded in genetic maps, their documentation and annotation. The database management system is robust and has continuously been Sybase. In this brief review we provide an introduction to the database as a functional genomics tool and new accesses to the data: 1) probe tables by bin location 2) BLAST access to map data 3) cMap, a comparative map graphical tool.


**Comparative and Functional Genomics**
*Comp Funct Genom* 2002; **3**: 128–131.
Published online 14 March 2002 in Wiley InterScience (www.interscience.wiley.com). **DOI:** 10.1002/cfg.157

**Conference Review**

### MaizeDB – a functional genomics perspective

Mary Polacco[1,2]*, Ed Coe[1,2], Zhiwei Fang[2], Denis Hancock[2], Hector Sanchez-Villeda[2] and Steve Schroeder[2]

[1] *USDA-ARS*
[2] *Department of Agronomy, University of Missouri, Columbia, MO 65211 USA*

**Correspondence to:*
*203 Curtis Hall, Dept Agronomy, University of Missouri, Columbia, MO, 65211 USA.*
*E-mail: PolaccoM@missouri.edu*

Received: 11 February 2002
Accepted: 13 February 2002

### Abstract

**MaizeDB (http://www.agron.missouri.edu/) has existed since the early 90's as a genome-specific database that is grounded in genetic maps, their documentation and annotation. The database management system is robust and has continuously been Sybase. In this brief review we provide an introduction to the database as a functional genomics tool and new accesses to the data: 1) probe tables by bin location 2) BLAST access to map data 3) cMap, a comparative map graphical tool. Copyright © 2002 John Wiley & Sons, Ltd.**

**Keywords:** **Database; maps; genome; phenotypes; agronomic traits; BLAST; comparative maps**

It is in the annotation of genetic maps and related objects that MaizeDB includes a substantial amount of functional information at both the molecular (gene product), but also at the whole plant (phenotype and agronomic trait) level. Gene product assignments are either empirically (with reference to a literature publication) or computationally (based on sequence similarities) determined. Phenotypic information derives from the substantial mutant collections (which are available from the Maize Genetics Cooperative Stock Center in Illinois) and the information they have revealed about maize physiology, development, evolution and map locations. Other phenotypic genome data are imported from researchers and from published studies on the inheritance of agronomic traits and associated quantitative trait loci (QTL). All data sources are credited to providers, and with reference to the published literature. Over 0.5 million links to external databases are represented. (Note: links to the mirrored sequence databases, GenBank, EMBL, and DDBJ are counted as a single link).

MaizeDB phenotype records reference one or more of 95 body parts (for example leaf, endosperm, pericarp); one or more of 46 developmental stages (belonging in turn to one of three recognized staging systems); one of 566 traits (for example pigmentation, response to corn earworm); one or more of 121 metabolic pathways (for example, lysine biosynthesis); one or more of 6908 genetic and QTL variations; and one or more of 21 000 stocks, many with availability information (notably the Maize Genetics Cooperation Stock Center). There are links to GRIN, the US Germplasm Resource Information Network for germplasm evaluations and availability. Genetic and QTL variations are linked to one of over 11 000 mapped loci, which are linked to raw and summarized map data, to any of the 900 maps for maize, to gene products and to sequence databases (GenBank, EMBL, DDBJ, SwissProt). Every effort is made to use standards in trait, body part and developmental stage descriptors; efforts include collaboration with the Plant Ontology Consortium (POC), modeled on the Gene Ontology (GO) and consulting with the maize Crop Germplasm Committee. The POC was presented at the PAGX meeting (see The Plant Ontology Consortium, in this issue). In addition, MaizeDB has imported the classification scheme of the Enzyme Commission and the ProSite vocabulary for motifs and provides links back to the Expasy site for details.

To access functional data from a genomics perspective, a user needs to keep in mind that 1)

Copyright © 2002 John Wiley & Sons, Ltd.

genetic maps are a focus, 2) there are multiple maps, and 3) bins maps integrate the many genetic maps. Knowing about bins is akin to a traveler having some knowledge of geography when deciding which road-maps to take on a trip. Bins maps are a low resolution integration of over 11 000 loci that are ordered onto any of some 900 maps; they are made by MaizeDB staff. Bins map locations are provided on Locus, Probe and Trait records. Thus Google, Name and Focused queries, referred to briefly below, will access the bin value(s), when they provide look-ups of Locus, Probe and Trait records. In addition, as a result of a collaboration with the Maize Mapping Project (Missouri), users may now do BLAST on maize sequences, and obtain bins and other map coordinates and links to external databases (kadath.agron.missouri.edu). This utility was first unveiled at the PAGX meeting in San Diego. It is a response to cooperator requests and suggestions.

'Google', indexes the database 1–2 times monthly, and kindly permits full-text searches by site. We also take advantage of a new Sybase tool, which uses a more robust search style, and is based on the Verity language. The Sybase tool only searches text fields within the database while Google searches all files on the server. Full text searching, for novice users, as well as jaded experts, is often the fastest access to approximate map location for an object, or the locus symbol for an object of interest. To look up all records relevant to a motif, for example 'P450' returns some 360 items when searching by 'Google'; a query on 'opaque endosperm' returns over 1100 records. Google returns both class of information (Locus, Phenotype, Reference, etc), the name of the object and a few lines from each record. Other methods for finding information include forms, which return lists of names or symbols of the category being searched, but not any other details from the page. For example, typing the phenotype 'opaque endosperm' onto the focused Locus query form retrieves a selection list of 249 locus symbols and full names.

New look-ups are being developed that retrieve tables to browse, while also simplifying the search strategy. Probe and clone searches based on bins map coordinates now deliver, instead of a list of names, a dynamic table with map coordinates, locus

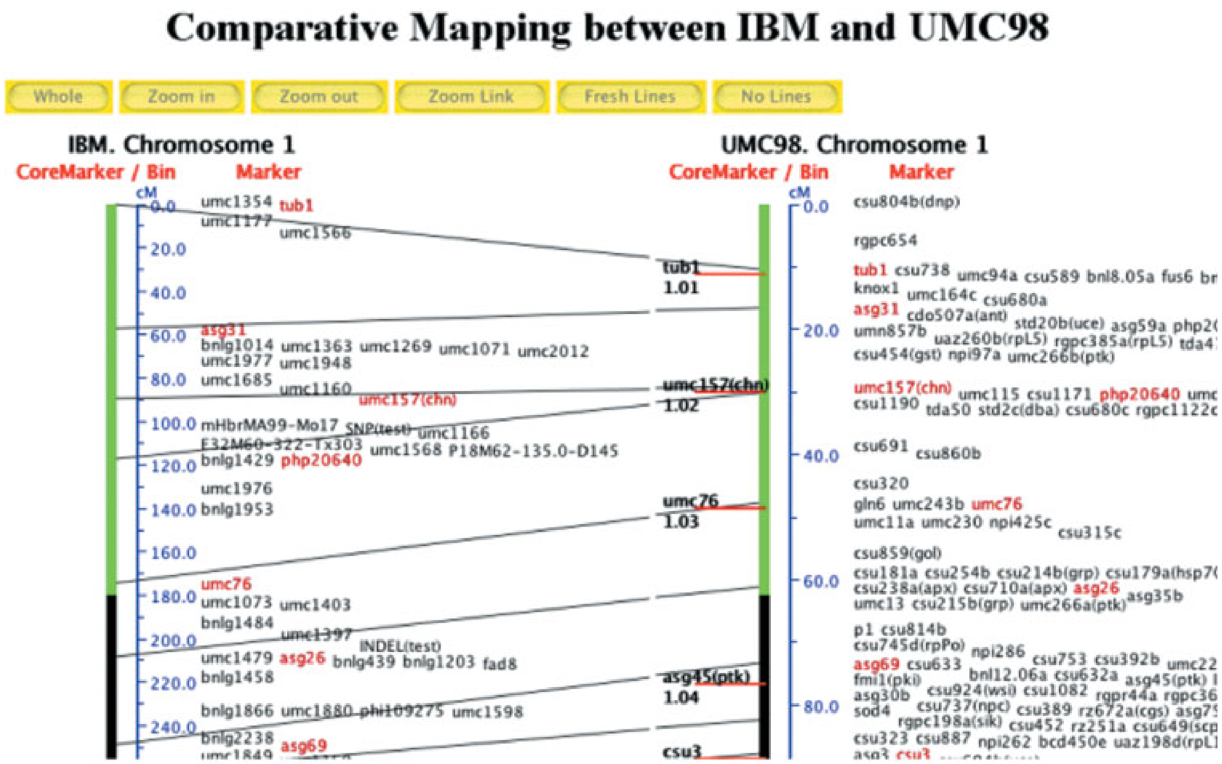

**Figure 1.** A cMap comparison of the IBM and UMC98 maps for maize chromosome 1

Copyright © 2002 John Wiley & Sons, Ltd. 

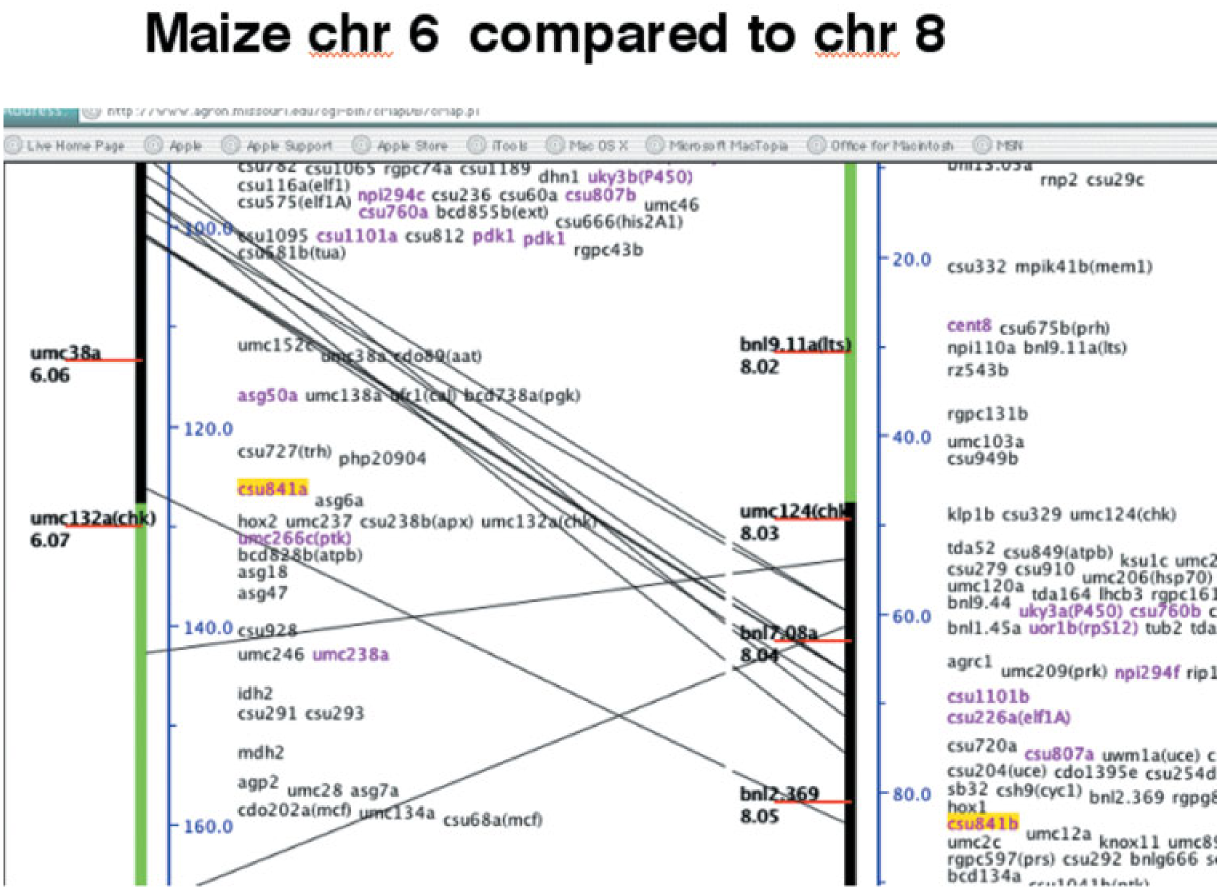

**Figure 2.** An intraspecies comparison using cMap; maize chromosome 6 with maize chromosome 8

and probe symbols, links to MaizeDB for details, links out to GenBank, to CUGI (**http://www.genome.clemsom.edu/**) for BAC contig information and clone requests, to TIGR (**http://www.tigr.org/**) for EST assemblies and comparative sequence information, and to ZmDB (**http://www.zmdb.iastate.edu/**) for EST assemblies and clones.

A main point of the bins map is to create a foundation for map-based activities, such as candidate gene cloning, or selection of nearby markers for agronomic traits important to a breeding project. Bins do not, however, provide the resolution required for map-based cloning of a candidate gene for a mapped trait, for example drought tolerance. Bins are 10–20 cM regions along the chromosome; the boundaries of each are defined by a set of core RFLP markers on the University of Missouri (UMC) maps. Of the 11 000 mapped loci, only subsets of these appear carefully ordered on any map of some 90 or so for each linkage group. Well-ordered maps typically include fewer than 2000 loci and there is less than 20% overlap of mapped loci among more extensive maps such as the BNL (Brookhaven National Laboratory) and UMC (University of Missouri) maps (*Maize Genetics Cooperation Newsletter* (MNL) 1997 vol. 71, **http://www.agron.missouri.edu/mnl/71/maizedb71.html**). The resolution of a bin, 10–20 cM, approaches the resolution of classical phenotype and QTL maps. Early RFLP genetic maps provide some 10-fold higher resolution; a resolution of 0.05 cM is predicted for the IBM (Inter-mated B73 × Mo17) genetic maps, where the mapping population is a recombinant inbred mapping population of some 300 individuals (**http://www.maizemap.org/**) that represent a 3-fold expansion, i.e. equivalent to 1800 meiotic products.

A comparative map graphical utility, cMap,

Copyright © 2002 John Wiley & Sons, Ltd. 

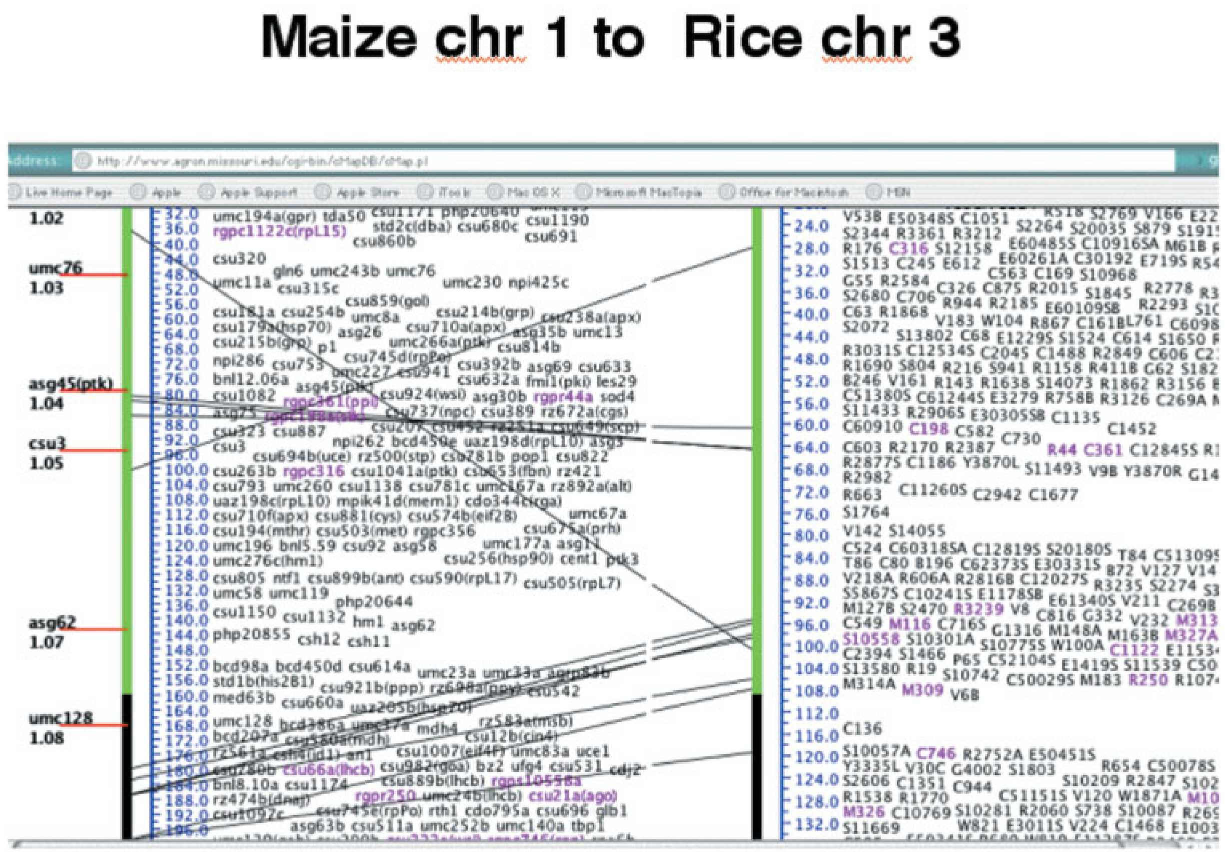

**Figure 3.** An interspecies comparison using cMap; maize chromosome 1 with rice chromosome 3

provides access to higher resolution, comparing two maps at a time. One use has been selection of SSR markers for an experiment which involves resolution greater than a bin, and where all SSR need to be available. These markers are currently on at least four sets of maps, with some overlap of SSR and RFLP on each of the sets and all are accessible to cMap. Options permit the user to select maps and to compare two maps at a time. Scrolling and zooming are enabled. Connecting lines and color coding designate loci 1) mapped by the same probe, or sequence, e.g. loci *csu1151a* and *csu1151b*, or 2) with the same name, for example in comparing 2 different maps for chromosome 1 (Figure 1). Clicking on a locus reveals a pop-up with a few details and links to MaizeDB and other data repositories. Both intra- and inter- species comparisons are supported (Figures 2 and 3). Maps currently accessible for comparison include 10 linkage groups each for 4 SSR based maps, and the BNL 96, UMC 98, and RGP 2000 maps. We anticipate addition of QTL maps, sorghum genetic maps, the Cornell rice genetic maps, and an updated RGP map over the year, as well as an updated IBM map with RFLP and SSR markers. These data have been made available to Gramene
(a comparative grasses map database, **http://www.gramene.org/**) and the NCBI-Genomes division, using a public SQL-data port to MaizeDB. This software appropriates display source code from GIOT, developed by the Rice Genome Program (Tsukuba, Japan). It operates in a database environment. The cMap prototype was first revealed at PAGIX in 2001, and went on-line late spring 2001.

### Acknowledgement

Many persons contribute to MaizeDB functionality and data. We rely extensively on maize cooperators for data and advice, on the USDA-ARS and the NSF (DBI Plant Genome #9872655) for funding and the University of Missouri as our host.

Copyright © 2002 John Wiley & Sons, Ltd. 

